# Roles of the Excitation in Harvesting Energy from Vibrations

**DOI:** 10.1371/journal.pone.0141299

**Published:** 2015-10-23

**Authors:** Hui Zhang, Tianwei Ma

**Affiliations:** Department of Civil and Environmental Engineering, University of Hawai’i at Mānoa, Honolulu, Hawaii 96822, United States of America; Oregon State University, UNITED STATES

## Abstract

The study investigated the role of excitation in energy harvesting applications. While the energy ultimately comes from the excitation, it was shown that the excitation may not always behave as a source. When the device characteristics do not perfectly match the excitation, the excitation alternately behaves as a source and a sink. The extent to which the excitation behaves as a sink determines the energy harvesting efficiency. Such contradictory roles were shown to be dictated by a generalized phase defined as the instantaneous phase angle between the velocity of the device and the excitation. An inductive prototype device with a diamagnetically levitated seismic mass was proposed to take advantage of the well established phase changing mechanism of vibro-impact to achieve a broader device bandwidth. Results suggest that the vibro-impact can generate an instantaneous, significant phase shift in response velocity that switches the role of the excitation. If introduced properly outside the resonance zone it could dramatically increase the energy harvesting efficiency.

## Introduction

Harvesting energy from vibrations has been reckoned as a promising, enabling technology because of its potential in revolutionizing a variety of applications, such as sensing [1, 2], monitoring [3], in vivo powering of medical devices [4, 5], etc. The past two decades have witnessed considerable advances in vibratory energy harvesting, but challenges still remain to be addressed before the well-regarded potentials of this technology are turned into reality.

The objective of vibratory energy harvesting is to extract as much energy as possible from a vibrating body—the source. In doing so, a harvester must dynamically interact with the source, rendering a problem of targeted energy transfer between two systems [6, 7]. If one considers the problem in the entire parameter space, it is equivalent to the problem of global optimization, in which the harvested energy is the target for maximization. For devices whose responses to external excitations can be described in closed-form expressions, e.g. linear devices, the optimization problem can be solved exactly because the closed-form solutions span the entire parameter space [8]. For nonlinear devices, the optimization problem cannot be exactly solved because of the lack of closed-form representations of the responses. As a compromise, nonlinear devices/designs have been traditionally evaluated using the harvested energy as the performance indicator within a subset of the parameter space [9]. For example, the frequency responses obtained from the approximated, analytical solutions have been widely used for this purpose. If even the approximated solutions are not available, devices have been evaluated using numerical simulations and physical experiments that cover even smaller subspace of the parameter space. Caution must be exercised for such optimization based on a subset of the parameter space because the results may not be generalized. Not only may the conditions of the evaluations in the subset space favor a device over another, rendering an unfair comparison [10], the comparison itself also does not provide useful implication in the device performance outside the subset of the parameter space. For example, under a single-frequency excitation and provided that the stringent requirements on initial conditions are met, nonlinear devices perform well over a wider range of frequencies than linear devices [11–14]. Because of the inapplicability of the principle of superposition, however, such “broadband”performance does not indicate satisfactory performance if the excitation is of rich frequency content. On the contrary, nonlinear devices may not offer any benefit under a multi-frequency excitation [10, 15–17]. Thus, when the response cannot be described in closed form, such as the case of a nonlinear device, one has to return to the original problem of energy transfer.

There has been substantial work related to energy transfer between two systems. An account of targeted energy transfer most relevant to the current study can be found in Vakakis et el [18]. The effects of coupling strength on the performance of vibratory energy harvesting have been treated in [19, 20]. Nonlinear energy sinks with properly designed essential nonlinear components have been shown to be effective in harvesting energy from the vibrations of a free-free beam under a shock load [21]. Here we consider the case of continuous excitations. We show that although the excitation (or the host vibration) is the source of the energy to be harvested, the excitation can temporally behave as a sink for a limited time, during which energy flows from the harvester back to the excitation. When the closed-form representation of the device response cannot be found, manipulating the roles of the excitation to minimize the extent to which it acts as a sink appears to be a viable alternative to the theoretical optimal solution.

In this study, the well established phase changing mechanism of vibro-impact [22–24] was utilized to change the phase between the excitation and the velocity of an impacting seismic mass, leading to a wider bandwidth of operation. It was demonstrated that impacts caused an instantaneous phase shift in the velocity of the seismic mass in the device and thus change the instantaneous role of the excitation (e.g. from an instantaneous sink to a source). If introduced properly, the work provided by the excitation can be increased resulting in improved device performance. Note that such phase changing mechanism synchronizes the response with the excitation and thus, increasing the total mechanical energy provided by the excitation and the harvested energy. It is different than the phase changing mechanism introduced by synchronized switching [25, 26], in which switching synchronizes the output voltage and current, leading to maximized electromechanical force and increasing the amount of electricity generated while the mechanical energy from the excitation remains at the same level.

## Materials and Methods

### Theoretical analysis

Following the most common practice in this field, consider an energy harvester and assume that the energy is harvested through a resistive load. The governing equation for the system can be generally written as

$$m\ddot{x}+c_{m}\dot{x}+k\left(x\right)+\kappa y=F\left(x,t\right),$$ (1a)

$$\alpha \dot{y}+\beta y=\kappa \dot{x},$$ (1b)

where *x* denotes the displacement of the seismic mass *m*, *c*_*m*_ the linear mechanical damping coefficient, *k*(*x*) the restoring force, *κ* the linear electromechanical coupling coefficient, and *F*(*x*, *t*) denotes the general external force, including parametrical excitations. For inductive devices, *y*, *α* and *β* represent the induced current, the inductance of the winding and the total resistance, respectively. For capacitive ones, they represent the induced voltage, the capacitance of the piezoelectric element and the load conductance, respectively. In this study, the inductive devices under low-frequency excitations were considered. Thus, the electromechanical coupling, represented in Eq (1b), could be approximated as an algebraic relationship (because *α* < < *β*), i.e. $\beta y=\kappa \dot{x}$. Over a reasonable period of time *T*, e.g. the fundamental period of a periodic oscillation, the harvested energy is $E_{h}=c_{e}\;\int_{0}^{T}\;{\dot{x}}^{2}dt$ where *c*_*e*_ = *κ* ^2^/*β* represents the effective electrical damping. The total dissipated energy $E_{d}=\left(c_{e}+c_{m}\right)\;\int_{0}^{T}\;{\dot{x}}^{2}dt$ is compensated by the nett work of the excitation over *T*, i.e. $E_{f}=\int_{0}^{T}\;F\left(x,t\right)\dot{x}dt$.

**Fig 1:**
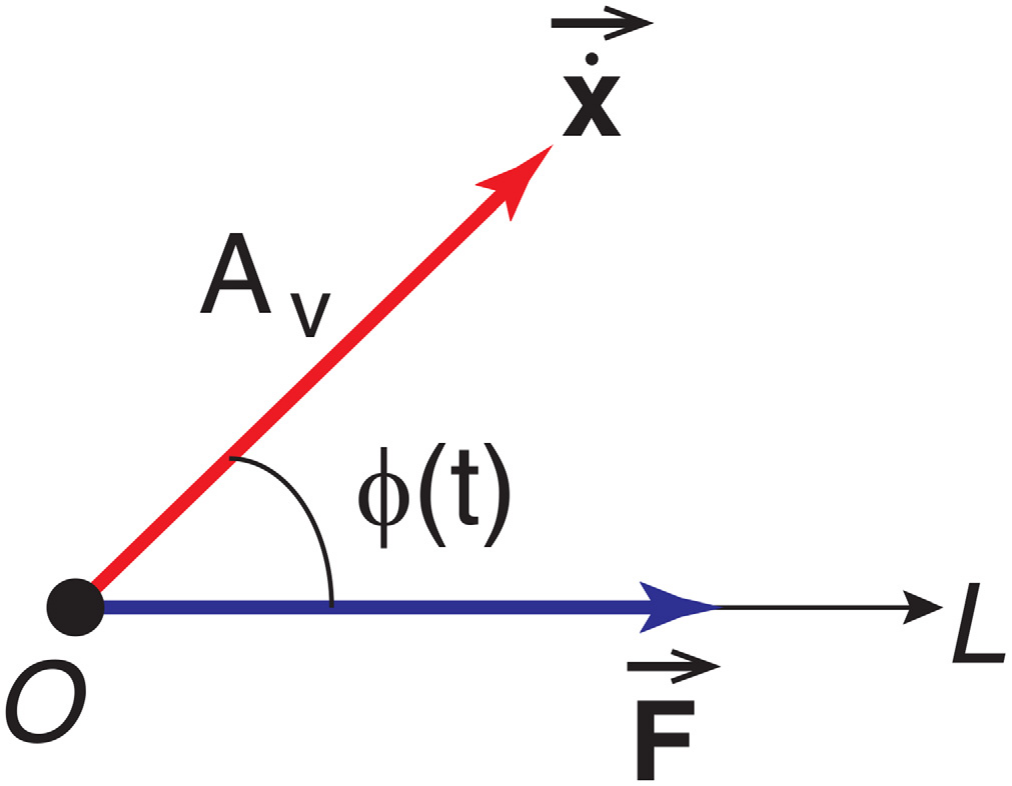
Vector representation of the excitation *F*(*x*, *t*) and the velocity of the response $\dot{x}\left(t\right)$.

The process of energy transfer between the excitation and the harvester can be analyzed using a generalized phase. As shown in Fig 1, taking the force direction as the polar axis $\vec{\text{OL}}$, the force *F*(*x*, *t*) can be represented by the force vector: $\vec{F}=\left(|F\left(x,t\right)|,0\right)$. The velocity $\dot{x}$ can be denoted as the projection of the vector $\vec{\dot{x}}=\left(A_{v},\phi \left(t\right)\right)$ on the polar axis $\vec{\text{OL}}$, where *A*_*v*_ is the magnitude of the velocity and *ϕ*(*t*) is defined as the generalized, instantaneous phase between $\vec{F}$ and $\vec{\dot{x}}$. For periodic cases, *A*_*v*_ can be defined as the amplitude of the response, therefore $\cos\phi (t)=\frac{\dot{x}}{A_{v}}\text{sgn}\left(F(t)\right)$. Note that for the case where multiple harmonics exist in the response, which could be due to nonlinear system response and/or a broadband excitation, the generalized phase *ϕ*(*t*) is a nonlinear function of time. However, if there is a dominating frequency *ω*_*d*_ in the response, a linear approximation may be possible, i.e. *ϕ*(*t*) ≈ *ω*_*d*_ *t*+*ϕ*_0_, *ϕ*_0_ being a constant phase lag. The instantaneous power of the excitation is $p_{f}\left(t\right)=\vec{F}\cdot \vec{\dot{x}}=\left|F\left(x,t\right)\right|A_{v}\text{cos}\phi \left(t\right)$. The role of the excitation in the energy transfer process is reflected by the instantaneous phase, cos *ϕ*(*t*) > 0 indicating that excitation works as an energy source to the device and cos *ϕ*(*t*) < 0 indicating that excitation draws energy from the device, acting as a sink. The nett work of the excitation can be rewritten as $E_{f}=\int_{0}^{T}\;\left|F\left(x,t\right)\right|A_{v}\cos\phi (t)dt=|W^{+}\left|-\right|W^{-}|$ where *W* ^+^ and *W* ^−^ denote the positive and negative work over *T*, respectively. The sum of the absolute values of the positive and negative work represents the actual “effort”of the excitation. It has been shown that under the global resonance condition [27], ∣*W* ^−^∣ = 0, the total effort of the excitation is completely converted into the useful work. Under a non-resonant condition, ∣*W* ^−^∣ ≠ 0, thus ∣*W* ^+^∣ − ∣*W* ^−^∣ < ∣*W* ^+^∣ + ∣*W* ^−^∣, only part of the total effort of the excitation contributes to the useful work. Therefore, the performance of the harvester can be evaluated using the coupling efficiency defined as

$$\eta =\frac{|W^{+}\left|-\right|W^{-}|}{|W^{+}\left|+\right|W^{-}|}=\frac{\int_{0}^{T}\;\left|F\left(t\right)\right|\cos\phi (t)dt}{\int_{0}^{T}\;|F\left(t\right)||\cos\phi (t)|dt}.$$ (2)

For a given excitation, adjustment of the phase can lead to changes in the work of the excitation and the coupling efficiency *η*, i.e. Δ*E*_*f*_ = Δ*W* ^+^ − Δ*W* ^−^ and $\Delta \eta =\frac{2(\Delta W^{+}|W^{-}|-\Delta W^{-}\left|W^{+}\right|)}{\left(|W^{+}|+\Delta W^{+}+\left|W^{-}\right|+\Delta W^{-})(|W^{+}\left|+\right|W^{-}|\right)}$. There are five general possibilities:

- Case I: Δ*W* ^+^ > 0 and Δ*W* ^−^ < 0, then Δ*η* > 0 and Δ*E*_*f*_ > 0;
- Case II: Δ*W* ^+^,Δ*W* ^−^ < 0 and $\frac{\Delta W^{+}}{\Delta W^{-}}<1$, then Δ*η* > 0 and Δ*E*_*f*_ > 0;
- Case III: Δ*W* ^+^,Δ*W* ^−^ < 0 and $1\leq \frac{\Delta W^{+}}{\Delta W^{-}}<\frac{W^{+}}{W^{-}}$, then Δ*η* > 0 and Δ*E*_*f*_ ≤ 0;
- Case IV: Δ*W* ^+^,Δ*W* ^−^ < 0 and $\frac{W^{+}}{W^{-}}\leq \frac{\Delta W^{+}}{\Delta W^{-}}$, then Δ*η* ≤ 0 and Δ*E*_*f*_ < 0;
- Case V: Δ*W* ^+^ < 0 and Δ*W* ^−^ > 0, then Δ*η* < 0 and Δ*E*_*f*_ < 0.

Note that when the coupling efficiency increases, Δ*η* > 0, the total harvested energy increases because of the reduction of the negative work (Case I, II), except when the reduction of the positive work is more significant (Case III). When the coupling efficiency reduces, Δ*η* < 0, the total energy harvested reduces (Case IV, V). Thus, if it is possible to adjust the phase such that the positive work of the excitation is increased and the negative work is reduced, the harvested energy will increase.

### Experimental materials and model

**Fig 2:**
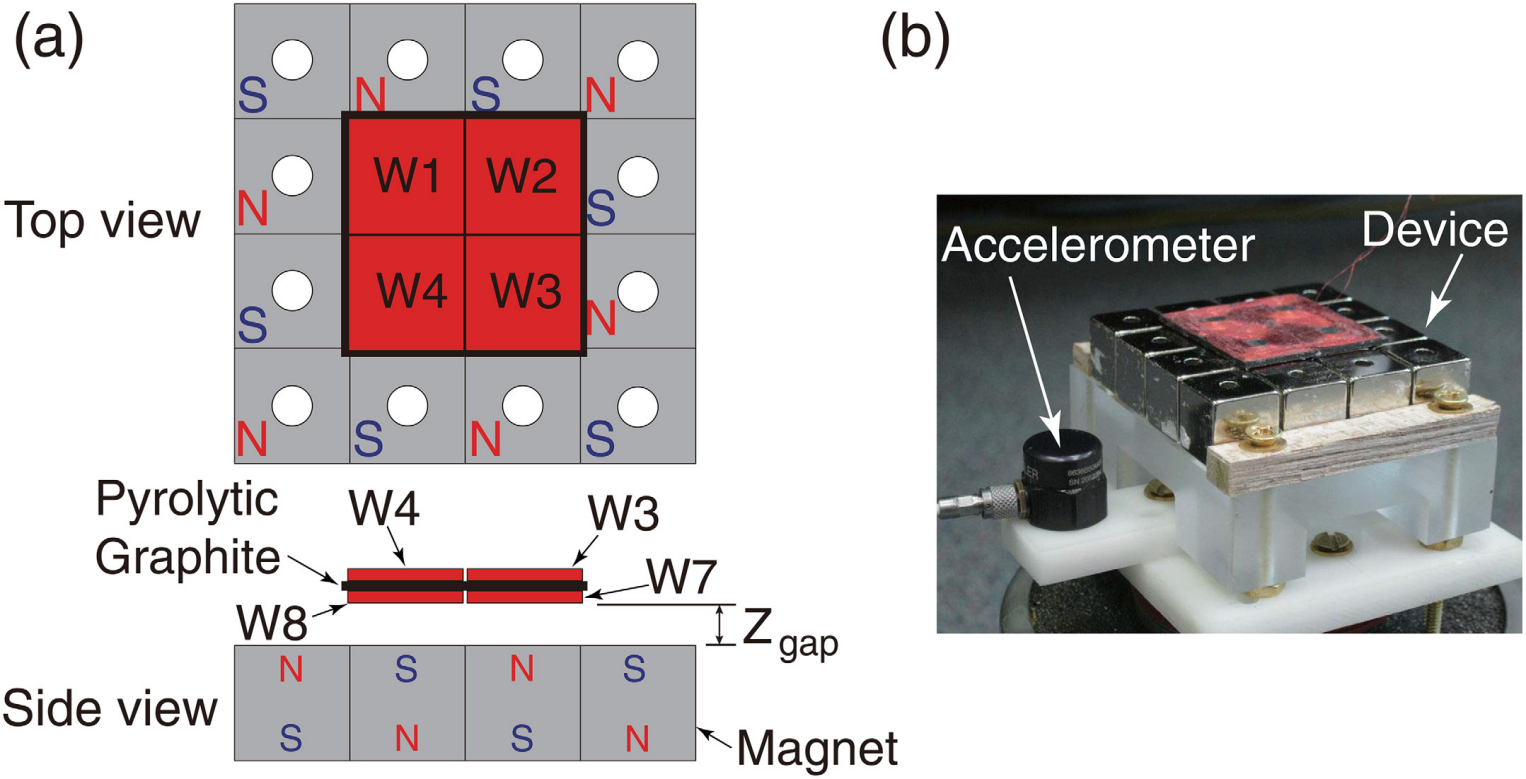
Prototype Device. (a) Schematic diagrams, and (b) the experiment apparatus (the shaker, the electrical load, and the oscilloscope are not shown).

In this study, vibro-impact was utilized to introduce sudden phase changes. An inductive device was constructed with a unilateral constraint to provide a nonlinearity that allows for a sudden change of the role of the excitation. The device (Fig 2) consisted of a diamagnetically levitated sheet of pyrolytic graphite (31.5 mm ×31.5 mm ×1 mm) [28–30] acting as a seismic mass. Eight identical windings were symmetrically attached to both sides of the graphite. The windings were fabricated using #44 AWG copper wire (each with 160 turns) and were connected in series giving a total resistance of 315.7 Ω. A resistive load, *R*_*L*_ was used as the electrical load to evaluate the device behavior. The mass of the pyrolytic graphite with the attached windings was 3.11 × 10^−3^ kg. Levitation was achieved over a sixteen-block array of cubic (12.7 mm) NdFeB magnets. The maximum magnetic flux density was measured to be 0.645 T on the surface of the magnet. At static equilibrium, the air gap between the levitated component and magnet was *z*_*gap*_ = 0.25 mm. Because the air gap was much smaller than the length scale of the pyrolytic graphite sheet, the squeeze-film effect was considerable [31]. In order to reduce such effect, a through hole of 3.23 mm in diameter was drilled in the center of every magnet, parallel to the magnetization direction. The linear fundamental frequency and mechanical damping ratio of the device were identified from the free-vibration tests to be *ω*_0_ = 16.1 Hz and *ξ*_*m*_ = 9.66%, respectively. The device was tested with a shaker (Model LW-126-13, Labworks Inc.) as the excitation source. Measurements were obtained using an oscilloscope (Model DPO 3040).

Because of the unilateral constraint, mechanical impacts occurred when the vibration reached the prescribed amplitude. The dynamics involving impacts were represented by the following equations [32]

$$\begin{array}{cc} \ddot{z}+2\omega_{0}\xi \dot{z}+g-\frac{F_{d}\left(z\right)}{m}=f\left(t\right), & z>-z_{gap}\\ \dot{z}\to -e_{k}\dot{z}, & z=-z_{gap}, \end{array}$$ (3)

where *e*_*k*_ is the coefficient of restitution, identified to be *e*_*k*_ = 0.6 in this study. The gap between the floating seismic mass and the magnets, *z*_*gap*_, dictated the occurrence of mechanical impacts. The instantaneous phase shift of the velocity was represented using the phase *ϕ*(*t*), i.e. cos *ϕ*(*t*) → −*e*_*k*_ cos *ϕ*(*t*), which shows that after the impact, the phase *ϕ*(*t*) was shifted dramatically indicating a change of direction of the velocity. The total damping included the mechanical damping (*ξ*_*m*_) and the equivalent electrical damping (*ξ*_*e*_), i.e. *ξ* = *ξ*_*m*_+*ξ*_*e*_. Symbol *g* denotes the gravitational acceleration. The diamagnetic force *F*_*d*_(*z*) was determined experimentally to be *F*_*d*_(*z*) = (9.2823 × 10^6^ *z* ^2^+3.3979 × 10^4^ *z*+32.79)^−1^.

## Results and Discussion

**Fig 3:**
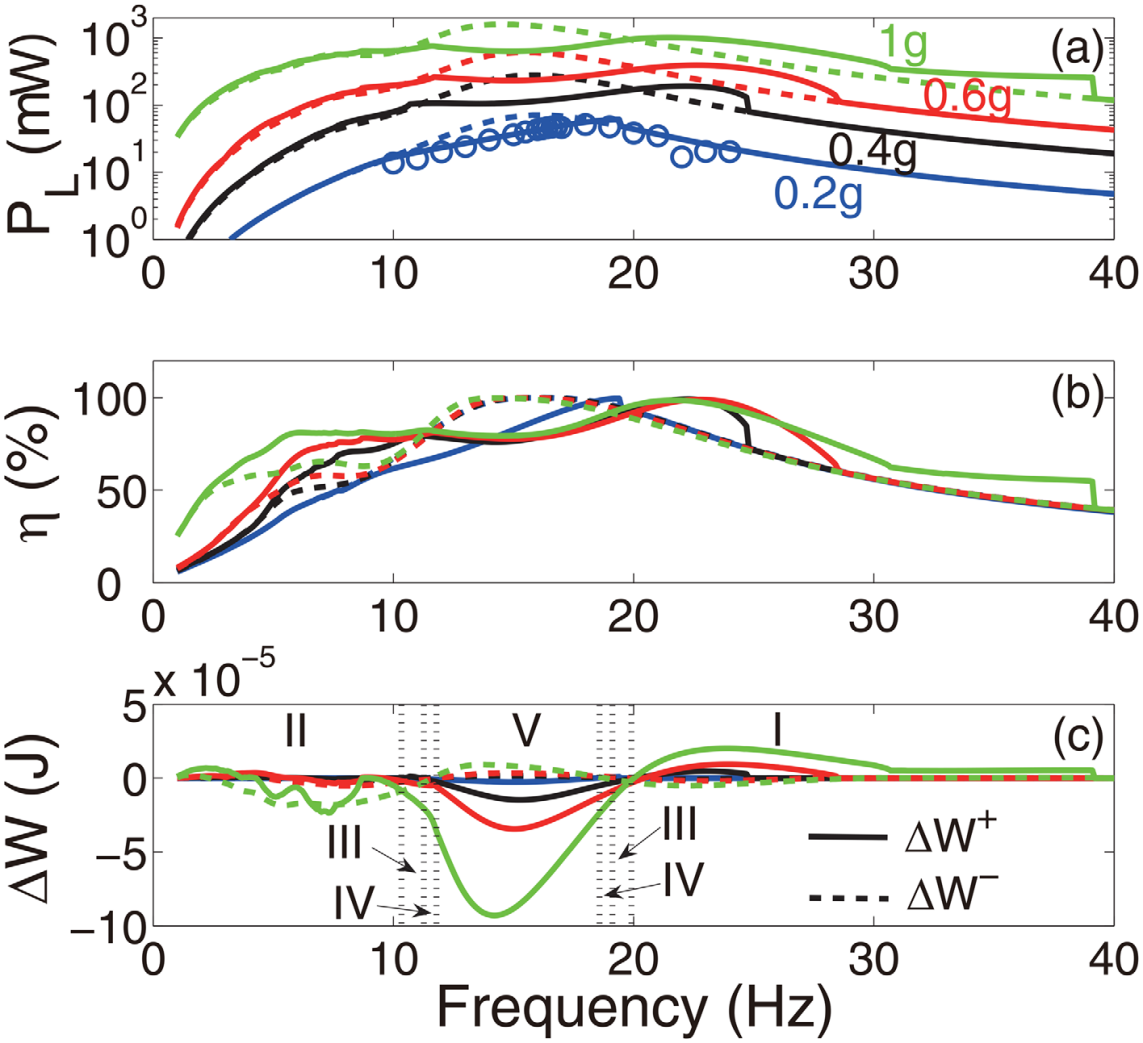
Results obtained under single-frequency excitations (*R*_*L*_ = 1 kΩ). (a) The average power *P*_*L*_ delivered to the resistive load, (b) the coupling efficiency *η*, (c) changes in the positive and negative work of the excitations Δ*W*. Lines: numerical results, circles: experimental results. In (a) and (b), solid lines: the prototype device, dashed lines: the artificial system without the amplitude constraint.

**Fig 4:**
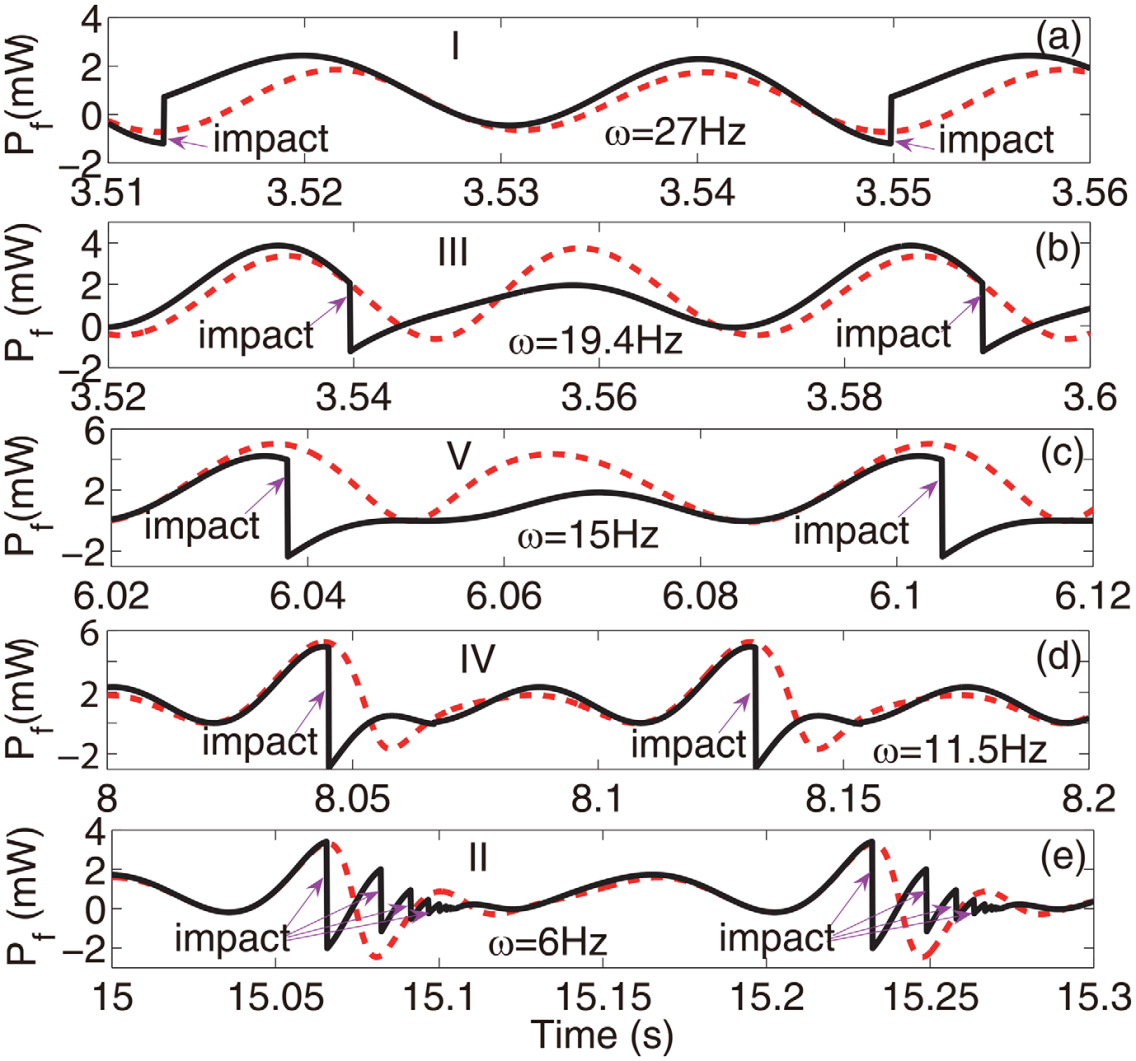
The instantaneous work of single-frequency excitations (*A*_*e*_ = 1 *g*, *R*_*L*_ = 1 kΩ) for the five difference cases (i.e. I–V). Solid lines: the prototype device. Dashed lines: the artificial system without the amplitude constraint.

Fig 3 summarizes the results from the numerical and experimental studies conducted for single-frequency excitations, i.e. *f*(*t*) = *A*_*e*_ cos *ωt*. The numerical simulations were conducted using the Dormand-Prince method (MATLAB^©^ ode45). Four excitation levels, *A*_*e*_ = 0.2 *g*, 0.4 *g*, 0.6 *g*, 1.0 *g*, were considered. Because of the limitations of the experiment apparatus, only the excitation level of *A*_*e*_ = 0.2 *g* was used for the experiments. For all the experimental cases, the resistive load was fixed at *R*_*L*_ = 1 kΩ, corresponding to an electrical damping ratio of *ξ*_*e*_ = 18%. The results for the artificial case without the amplitude constraint and thus without impacts were obtained using the theoretical model, Eq (3). They are also shown for comparison. The excellent agreement between the numerical and experimental results shown in Fig 3(a) demonstrates the validity of the theoretical model. The instantaneous changes of the phase due to the impacts were favorable outside the resonant zone of the device, as shown in Fig 3(a) and 3(b). The correlation between the coupling efficiency and the harvested energy appeared to be consistent, i.e. reducing the coupling efficiency led to the reduction of the harvested energy and increasing the harvested energy led to the increased efficiency, as predicted respectively by Cases IV and V and Cases I and II discussed previously. In some small regions, 10.4–11.2 Hz, 19–19.9 Hz, Case III dominated. Fig 3(c) illustrates the changes of the absolute values of the positive and negative work of the excitation due to the phase changes. When the frequency of the excitation was much lower than that of the device, the positive and negative work simultaneously decreased, corresponding to Cases II, III, and IV. For excitations with frequencies close to the fundamental frequency of the device, the positive work reduced and the negative work increased, corresponding to Case V. At higher excitation frequencies, Case I dominated, i.e. the negative work was reduced and the positive work was increased. Fig 4 shows the time histories of the instantaneous work of the excitation for the five different cases (i.e. I–V) under a single-frequency excitation of different frequencies with a fixed amplitude *A*_*e*_ = 1 *g*. It is noted that for the type of device architecture considered, mechanical impacts occur only at times when the seismic mass is moving away from the rest position. When the period of the excitation is shorter than the natural period of the device, there is generally a phase lag of the response velocity of $0<\Delta \phi <\frac{\pi }{2}$. The excitation does positive work as the seismic mass immediately moves away from the rest position. The positive work then gradually changes to negative because of the frequency mismatch. The exact size of the region within which the work is positive depends on the phase lag. A minimal distance between the constraint and the rest position is required to guarantee that impacts would occur in the region of negative work, corresponding to Case I. As the period of excitation approaches the natural period of the device, the region of the positive work is enlarged, requiring a larger distance prior to the impact, otherwise Case III, IV or V could dominate depending on the parameters. At resonance, the impacts limit the amplitude of vibration of the seismic mass, thus reducing output power, corresponding to Case V. When the period of the excitation is longer than the natural period of the device, the phase lag of the response velocity is $-\frac{\pi }{2}<\Delta \phi <0$. The excitation does positive work during the whole course of the seismic mass moving away from the rest position. Thus, impacts occur in the region of the positive work. Cases II–V could dominate depending on the parameters.

**Fig 5:**
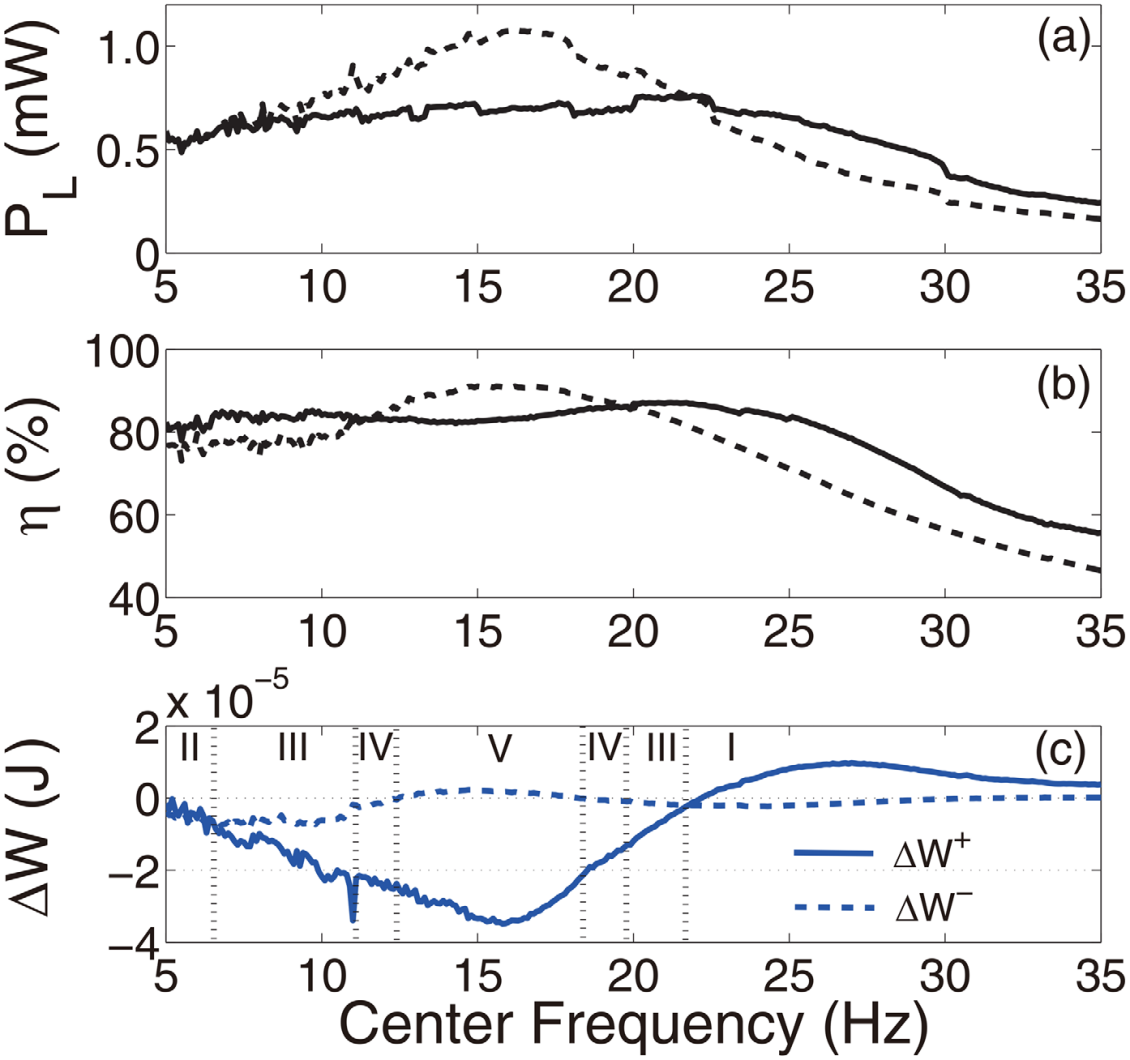
Numerical results obtained using band-limited excitations (*R*_*L*_ = 1 kΩ, bandwidth = 10 Hz, and PSD = 0.005*π* *g* ^2^⋅Hz^−1^). (a) The average power *P*_*L*_ delivered to the resistive load, (b) the coupling efficiency *η*, and (c) the changes in the positive and negative work of the excitations Δ*W*. In (a) and (b), solid lines: the prototype device, dashed lines: the artificial system without the amplitude constraint.

Fig 5 shows the results from numerical studies for band-limited excitations with a bandwidth of 10 Hz and a flat power spectral density of 0.005*π* *g* ^2^⋅Hz^−1^. The center frequencies of 5–35 Hz were considered. The results resembled those of the single-frequency excitations. If the energy of the excitation was concentrated on higher frequencies, it appeared to be guaranteed that the impact induced instantaneous phase change would improve the performance (Case I). Band-limited excitations with different bandwidths (5–15 Hz) were also considered for the same range of center frequencies. The results demonstrated qualitatively the same trend. They are not shown for brevity.

## Conclusions

Although the excitation is ultimately the source of the harvested energy, the extent of the excitation acting as a sink is critical to energy harvesting efficiency because it represents the wasted “effort”of the excitation not converted to harvested energy. The contradictory roles of the excitation were shown to be dictated by the mismatch between the properties of the excitation and the device. The degree of such mismatch can be represented by the generalized phase angle between the device response and the excitation. As demonstrated, an instantaneous phase angle shift can be achieved via vibro-impacts and it changes the role of the excitation. If the impacts occur at appropriate time instants such that it switches the excitation from being a sink to being a source, the mismatch is temporarily eliminated and the harvested energy can be dramatically increased. For example, the power increased from 153.1*μ*W to 258.2*μ*W at *A*_*e*_ = 0.6*g* and *ω* = 26 Hz. For random excitations with a 10 Hz bandwidth, the vibro-impact was shown to dramatically increase the device bandwidth and the device efficiency was kept around 80% for center frequencies up to 20 Hz. Because real-world ambient vibrations are normally random with broad bandwidths, a panacean solution of eliminating the mismatch based on passive methods is unlikely to exist. Thanks to continuously improving ultra-low power sensing and switching technologies, methods in which the role of the excitation is monitored and adjusted would be more promising in bringing vibratory energy harvesting to fruition.
